# The ethnobotanical heritage of Lotkuh, a high-altitude tribal haven of Chitral, the Eastern Hindu Kush, Pakistan

**DOI:** 10.1186/s13002-024-00687-8

**Published:** 2024-05-19

**Authors:** Hafiz Ullah, Lal Badshah

**Affiliations:** Phytoecology Lab, Department of Botany, University of Peshawar, Peshawar, 25120 Pakistan

**Keywords:** Ethnobotany, Chitral, Hindukush, Endemic flora, Traditional knowledge

## Abstract

**Background:**

In northwestern Pakistan, Lotkuh is a high-altitude terrain nestled within the eastern Hindu Kush region. Enclaved by towering peaks and harboring a unique culture, the region mirrors the geographical and cultural diversity of Pakistan. In this geographically isolated region, a treasure trove of ethnobotanical knowledge unfolds through generations of interaction between the inhabitants and indigenous plants, resulting in a profound understanding of the plant uses in nutritional, medicinal, cultural, and ritual contexts. Thus, the study seeks to gather, analyze, and document the indigenous knowledge of plant utilization of the distinct tribal culture.

**Methods:**

Through semi-structured questionnaires, inventory interviews, and participatory workshops, data were collected by engaging a cohort of 120 local respondents. The collected data were then classified into nine distinct use categories, following which quantitative indices were calculated.

**Results:**

The research identified a total of 150 plant species spanning across 59 different families and categorized them into 9 distinct usage groups. Among these, *Astragalus oihorensis, Astragalus owirensis, Cicer nuristanicum, Geranium parmiricum,* and *Rochelia chitralensis* stand out as novel species with distinctive applications. Notably, medicinal use garnered 600 reports, while animal feed, veterinary applications, human consumption, and toxicity recorded 500, 450, 425, and 104 reports, respectively. Informant consensus was high ranging between 0.8 and 0.9 with most agreement on human food and animal feed category. *Platanus orientalis* and *Juglans regia*, with RFC 0.91, were the most cited. The Family Importance Value (FIV) of Juglandaceae and Platanaceae, each with an FIV of 0.91, and Capparidaceae with an FIV of 0.83 indicate the intricate role the families play.

**Conclusions:**

In this study, we explore 150 ethnobotanical species, uncovering novel entries within ethnobotanical literature. Among these, several species showcase unique uses previously undocumented in Pakistani literature. Our research sheds light on the intricate interaction between plants and the distinct cultural landscape of the Lotkuh region.

## Background

The people of the eastern Hindukush region in northwestern Pakistan have a long history of using wild plants to sustain their life amidst the rocky, narrow valleys of the snow-capped mountains. The plants of the high-altitude mountain ecosystem have provided the dwellers with everything from food to fodder, medicine to firewood, and cultural raw materials. Since they have always lived in the highlands, the people of northwestern Pakistan have a much deeper relationship with wild flora to satisfy their everyday essentials [1]. Inhabitants of the Hindukush region along the Pakistan-Afghanistan border have a distinctive culture, which includes a unique food system based on wild food plants and the use of plants for both health and economic well-being [2].

Throughout the ages, man has reaped direct and indirect advantages from the diverse array of wild flora. Wild plant resources have offered food to alleviate hunger, plant materials for textile production, timber for construction purposes, medicinal plants for therapeutic applications, and commodities for ceremonial merriments. Particularly, a considerable portion of the population in developing countries in recent days relies significantly on the income generated from the trade of wild plant products [3–5]. Furthermore, for economic motives, developed countries persistently procure high-value wild products such as mushrooms and medicinal herbs from their developing neighborhood [6]

Many people, especially in remote areas, rely on wild edible plants for their daily dietary needs [7, 8]. Wild plants in many cultures have a tremendous impact on human life, primarily because of their nutritional importance. Today, it is undeniable that wild plants are used as a source of nutritional supplements [9]. For generations, rural communities worldwide have incorporated natural herbs into their diets through traditional foods, herbal juices, teas, and beverages. It is an established truth that medicinal plants are a significant source of newly discovered drugs [10]. Rural communities have been consuming a diverse range of wild foods for an extended period, with the preparation techniques being handed down from one generation to the next [11].

Traditional ethnobotanical knowledge (TEK) is inherited from one generation to the other as a subsequent part of the indigenous knowledge heritage of the rural communities [12]. The knowledge of traditional plant utilization is changing because of sociocultural changes in local communities, and ethnobotanical knowledge is being seriously hampered by industrialization and globalization. Because of this, only roughly 20 edible plant species account for about 90% of our daily food, even though there are around 20,000 nutritious species of plants in the wild. In most of Asia, the local traditional knowledge of plant resources is on the brink of extinction in rural areas, while in most urban settings no remnant is left behind [13]. Therefore, it is imperative to gather and document the traditional knowledge in rural areas, where it is practiced, to pass it to the next generation [14].

While ethnobotanical literature exists in Pakistan, the northwestern regions remain largely unexplored due to their remote and challenging accessibility. Lotkuh, situated in the secluded northwestern part of the country, stands distinct in geography, culture, and language from the rest of Pakistan. Its unique climate fosters a diverse flora, contributing to the country's botanical richness. Nestled among towering mountains, the inhabitants possess invaluable knowledge of plant usage for sustenance, medicine, cultural practices, and rituals. This pioneering endeavor marks the inaugural effort to document and conserve the ethnobotanical heritage of Lotkuh's ancient culture. Led by a native researcher, this initiative is a crucial response to the threats of cultural dilution and globalization.

## Materials and methods

### Geographical location of the study area

**Fig. 1:**
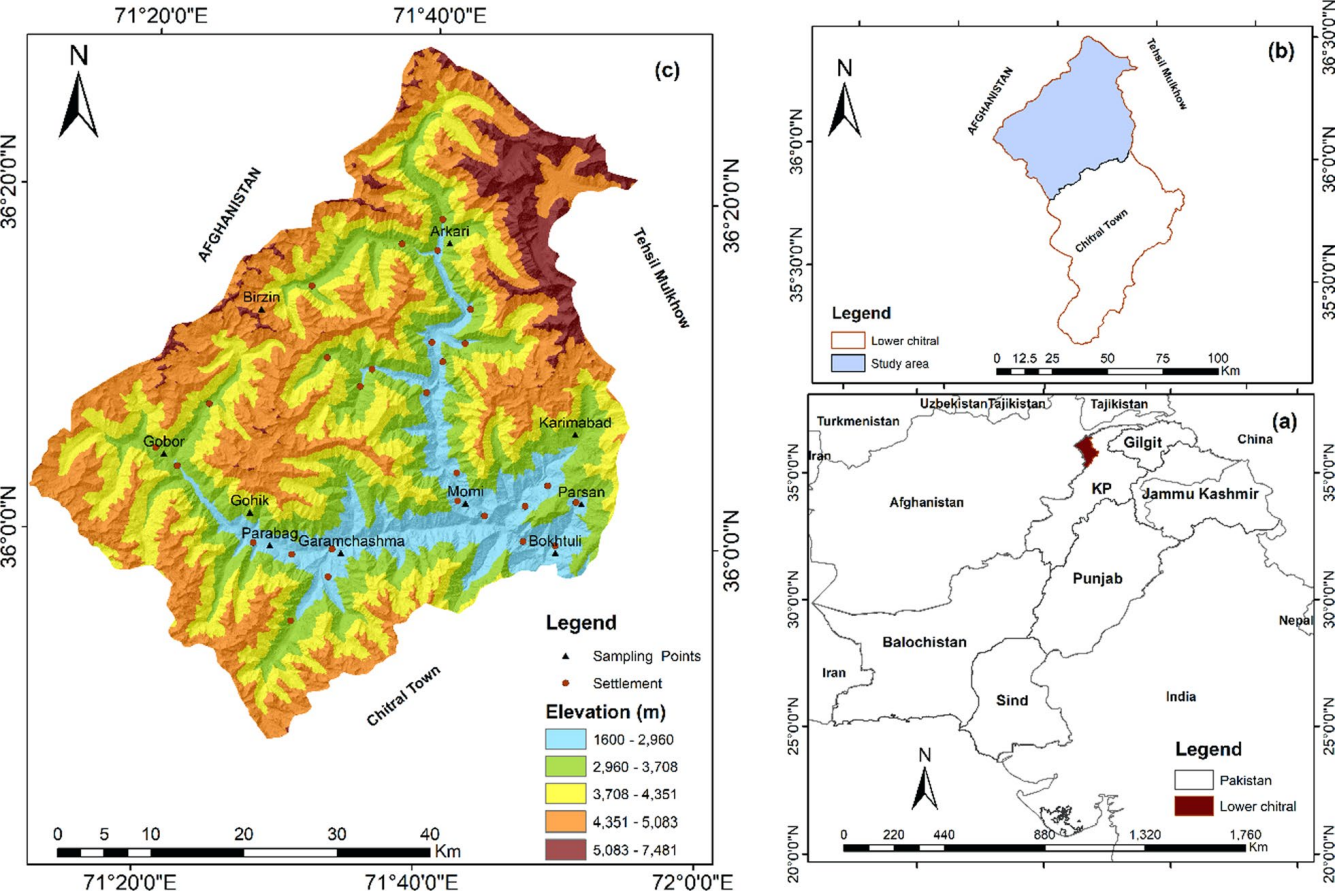
Geographical map, depicting the data points   in the study area (Lotkuh), Chitral

The Lotkuh region, which serves as the research area, occupies the northwest of Pakistan's Khyber Pakhtunkhwa province. Geographically, the study area is stretched between 35° 47ʹ 52ʺ to 36° 29ʹ 10ʺ north latitudes and 71° 11ʹ 52ʺ to 71° 54ʹ 42ʺ east longitudes. The valley has a rugged landscape and is located next to the Wakhan Corridor. The majestic Eastern Hindu Kush's vast biodiversity is reflected in the territory. The Terich Mir (7692 m a.s.l.), the highest peak in the Hindu Kush range, is located on the eastern of the research area. Throughout the year, these huge mountain ranges are blanketed in perpetual snow and glaciers. The elevation of the study area ranges from 1600 to 7000 m. The research area is subdivided into three sub-valleys, viz, Karim Abad, Arkari, and Garam Chashma. The geographic location is further illustrated in Fig. 1.

### Sociodemographic characteristics of the study area

The research area is culturally rich, and different local languages are spoken in the region. We selected 120 informants from the study area for our study. During the survey, detailed demographic data of the informants were acquired. The data included information on population, age, gender, ethnicity, language, religion, and occupation. The demographic details are provided in Table 1. The principle ecological features of the villages that serve as data points are illustrated in Table 2. Following are the major languages as the mother tongue of the born-in native inhabitants of the area.

**Table 1:** Geographical outline and sociodemographic profile of the respondents of each village visited during data collection

Village	Geography	Altitude	Population	Age	Sex	Ethnicity	Language	Religion	Occupation
Parsan	36° 02ʹ 28ʺ N 71° 51ʹ 17ʺ E	1650	716	40–70	M = 10 F = 4	Lotkuhi	Khowar	Sunni Ismaili	Hn, Tr, Sh, Fr
Bokhtuli	35° 59′ 16″ N 71°48′ 41″ E	3000	356	20–60	M = 4 F = 4	Ozhorek	Khowar	Sunni Ismaili	Wm, Pg, Ms, Sp
Karimabad	36° 61′ 32″ N 71°50′ 29″ E	3054	797	25–80	M = 8 F = 4	Parsaneko	Khowar	Sunni Ismaili	Cr, Pg, Hl, Ms,
Arkari	36°16′ 55″ N 71°40′ 15″ E	2900	1300	30–60	M = 5 F = 5	Arkareogh	Khowar Dari	Sunni Ismaili	Sh, PG, Hn, Fr
Momi	36° 2′ 17″ N 71°43′ 11″ E	2871	1095	40–80	M = 6 F = 6	Arkareogh	Lotkohiwar Khowar	Sunni Ismaili	Hl, Ms, Sp, Pg,
Garamchashma	35° 59′ 42″ N 71° 34′ 18″ E	2451		30–65	M = 10 F = 6	Prabegchi	Bashgali Yidgha	Sunni Ismaili	Hn, Tr, Pg, Fr
Parabag	35° 59′ 28″ N 71° 29′ 14″ E	2521	1,567	25–80	M = 9 F = 6	Lotkuhi	Lotkohiwar Khowar	Sunni Ismaili	Hl, Ms, Sp, Fr
Birzin	36° 2′ 10 ″ N 71° 27′ 55″ E	3214	837	30–77	M = 6 F = 7	Lotkuhi	Yidgha Bashgali	Sunni Ismaili	Hn, Tr, Pg, Fr
Gohik	36° 0′ 27″ N 71° 27′ 15″ E	3250	453	20–60	M = 6 F = 5	Lotkuhi	Yidgah Khowar	Sunni Ismaili	Hl, Ms, Sp, Pg
Gobor	36° 4′ 34″ N 71°21′ 4″ E	3319	1,028	30–80	M = 7 F = 2	Goborchi	Shekhnwar Dari	Sunni Ismaili	Fr, Sh, Pg, Hn

Hl = Healer, Fr = Farmer, Sh = Shepherd, Hn = Hunter, Tr = Teacher, Wm = Wildlife Manager, Pg = Plant Gatherer, Sp = Salesperson

Ms = Musician, Cr = Carpenter, M = Male, F = Female

**Table 2:** The principle ecological features of villages as data points for the ethnobotanical study

Village	Principle Ecological Features
Parsan	The region is classified as semiarid, relying heavily on melting avalanche water as its primary water source. The soil, predominantly fertile, consists of a combination of clay loam and sandy textures. It features alpine pastures and grazing lands and is renowned for hosting common bird species such as the *Alectoris chukar*
Bokhtuli	Situated in the foothills it is a dry, semi-arid region, featuring clay loam soil and hosts forests abundant with conifers and Quercus trees. It serves as a designated reserve for the national iconic animal, the Markhor (*Capra falconeri cashmiriensis*). Water is primarily sourced from nearby rivers
Karimabad	The village resides at a relatively high altitude, experiencing harsh winters. Snowfall serves as the primary source of precipitation. The soil composition comprises clay and stones. Within the village, glaciers are present, and vegetation is sparse, characterized by fewer trees and a prevalence of herbaceous species
Arkari	The village hosts flat terrain conducive to agricultural practices. Its predominantly grassland vegetation sustains the herders' livestock. Precipitation is mainly from snowfall and the melting of glaciers. The soil composition is primarily silty and sandy across most areas. The mountains are solid rocky formations with vertical cliffs and sparse tree species
Momi	This village sits upon an alluvial fan deposit and has long been inhabited by the Lotkuh people. The vegetation in this area is sparse, with most of the land dedicated to potato cultivation. Winter temperatures are harsh in this region
Garumchasma	This location is a narrow valley encircled by towering mountains. Throughout the area, there are hot Sulphur springs known as the “Hot Springs”. The soil here is rich and comprises a blend of clay and sand, ideal for agriculture
Parabag	From an ecological perspective, much of the village forms a river basin characterized by silty and clayey soil. The climate in this area is arid yet temperate, with early snowfall being a common occurrence. The average temperature is 16℃. The soil quality lends itself well to the commercial cultivation of peas and potatoes
Birzin	Situated on the western side of the study area, this village predominantly serves as a catchment area where snow avalanches settle. Sparse vegetation, primarily composed of herbs and edible wild food plants, adorns the landscape
Gohik	Situated on the western side of the study area, this village predominantly serves as a catchment area where snow avalanches settle. Sparse vegetation, primarily composed of herbs, adorns the landscape
Gobor	This region connects the western border with Afghanistan's Badakhshan province via the renowned Durah Pass. Cold weather prevails throughout the area, which features large and small glaciers that persist year-round. Betula and Salix are the dominant tree species. The soil, characterized by clay and boulders, is suitable for agricultural purposes

#### Khowar

Khowar is the lingua franca of this region and a major source of communication and researchers’ tool to collect data [15].

#### Yidgha

In the western sub-valleys of Lotkuh, Yidgha is the most spoken language. However, the population speaking Yidgha is smaller in comparison with other languages [16].

#### Lotkohiwar

A smaller group of people in the area speak Lotkohiwar, a language mostly related to Khowar but with minor differences in meaning and accent [17].

#### Sheikhan war

Some of the inhabitants aligned with the border of Afghanistan speak Sheikhan War. The people adhering to this language number very few. Gobor, a small village in proximity to Afghanistan, is the safe dwelling of the inhabitants who speak Sheikhan War [17].

#### Bashgali

Bashgali is a language spoken in the study area with limited comprehension among the local population. It distinctively differs from Khowar, which is the predominant language spoken by the majority in the region [18].

#### Dari

This language is considered non-native and was introduced to the valley due to the influence of Nasir Khisraw, the eleventh-century, poet, and philosopher. The charm and melody of the poetry attracted a considerable stratum of the society to adhere to it. One of his followers had come to this part of the region and got settled here permanently. The neighboring country, Afghanistan, was intervened by the then USSR, and as a result, a huge influx of migrants rushed to this area. Since Garam Chashma was easily accessible from Badakhshan, many Afghans chose to stay back and a few of the migrants are still living here. The people of Badakhshan communicated in Dari and gave birth to this language in the valley [19].

### Ethnicity

The study area is ethnically diverse, consisting of two distinct ethnic groups.

#### The Sunnis

These people practice the faith of Islam and are obedient to the Sharia of the Prophet, Mohammad. They live in harmony with the rest of the ethnic group.

#### The Ismailis

This ethnic group follows His Highness the Agha Khan as Imam (the spiritual leader whom they are obedient and followers). In most villages of Lotkuh the Ismaili ethnic group constitutes the majority population. This ethnic community coexists in perfect harmony with Sunnis, engaging in each other's rites, rituals, and cultural practices.

### Data collection

Data gathering in the current study relied on three methods: a semi-structured interview to collect data from the selected respondents; an inventory interview and a participatory workshop. Applying quantitative indices like Use Report (UR), Informants Consensus Factor (ICF), Relative Frequency of Citation (RFC), Use Value (UV), Fidelity Level (FL), Family Importance Value (FIV), Jaccard index (JI), and Pearson’s Pairwise comparison, the data were evaluated.

#### Semi-structured interviews

A total of 120 respondents were selected including males and females from diverse fractions of the society comprising educated professionals, farmers, local healers, nomads, and wildlife managers. Each informant first participated in a semi-structured interview to gather information about the common names of plants in the area, their uses, and the locations of collecting sites. The plant uses were then grouped into 9 distinct use categories (Table 3).

**Table 3:** Ethnobotanical use categories of plant taxa

S. no.	Use category	Code	Illustration
1	Medicinal	MED	Taxa used in folk medicine
2	Human Food	HF	Taxa that the villagers use as food
3	Animal Food	AF	Taxa serving fodder for livestock
4	Veterinary Uses	VU	Medicine for livestock and companion animals
5	Technologies	TECH	Technological instruments and craftworks
6	Timber	TIM	Plants that are used in building and carpentry
7	Firewood	FW	Fuel for domestic and commercial purposes
8	Symbolic	SYM	Used in festivities and cultural celebrations
9	Toxic	TOX	Unsafe and toxic for intake by humans

#### Inventory interviews

All plant specimens to be investigated were collected and shown to the respondents one by one to investigate the uses of these plant resources. The method is more useful when the local names are unknown to the investigator [20]. The informants were first asked the local names of the plants and their uses. These informants were recognized in their community for their extensive knowledge of the regional native flora.

#### Participatory workshops

A participatory workshop was conducted to validate the indigenous names and traditional uses of local plants. Members of the community were extended invitations to attend the workshop, during which the objectives were clearly outlined and demonstrated to them [21]. To conduct the workshop efficiently and make information gathering easier, the participants were separated into three groups. The groups were then shown the collected plant specimens and images. Thus, it was possible to establish the correct local names of the gathered specimens.

#### Field survey, plant collection, preservation, and identification

The field survey commenced in April 2021 and extended until December 2022. Villages and sub-valleys were the subjects of an intensive field survey. These sub-valleys and villages were the representatives of the total study area. Plant collection was made along with detailed data on habitat, life form, and morphology. The core of the identification was mostly based on Flora of Pakistan (http://www.efloras.org/), Tropicos (http://www.tropicos.org/), and the naming was confirmed by the World Flora Online (https://www.worldfloraonline.org/). Following the standard herbarium protocols, the collected specimens were pressed, dried, and mounted on herbarium sheets. The collected specimens were then submitted to the herbarium at the Department of Botany, the University of Peshawar, for reference.

#### Use report

Use reports are the information shared by the informants about the use of a species in a specific use category. Here the information taken from a participant (i) about the use of plant species (s) in a specific use category (u) is collected [22]. In a particular survey that comprises NS species (s_1,_ s_2,_……sNS) spreading over use-categories NC (u_1_, u_2_, u_3_,….uNC) and N informants…. (i_1_, i_2_,……iN), then the following formula can be employed to calculate use reports (UR);

1 $$UR={\sum }_{u={u}_{1}}^{uNC}{\sum }_{i={i}_{1}}^{iN}{UR}_{ui}$$

#### Informant consensus factor

When it is essential to scrutinize the homogeneity of the data provided by the informants in each use category involving a species, the informant consensus factor is used [23].

2 $$ICF=\frac{Nur-Nt}{\left(Nur-1\right)}$$

where ‘Nur’ denotes the number of use reports for a specific plant-use category and ‘Nt’ is the number of taxa associated with that use category. The value of ICF ranges between 0 and 1, where a value close to 1 indicates that relatively few taxa are used by a large proportion of the informants, and a value closer to 0 indicates that the informants differ in their use of taxa within a use category [24].

#### Relative frequency of citation

The most popular/used plants in an area are determined using this index. RFC value ranges from 0 (when it is not being used in that area) to 1 (if all the informants consider the plant species to be valuable). RFC illustrates the regional significance of each species without taking use categories into account [25]. To calculate (RFC), the following formula is used;

3 $$RFC=\frac{FC}{N}$$

where ‘FC’ is the frequency of citation, while ‘N’ represents the total number of informants participating in the study.

#### Use value

Use value refers to a taxon's relative importance as determined by its utility. The following formula is used to calculate the use value of a species [26].

4 $$UV=\frac{{\sum }_{1}^{n}{U}_{i}}{N}$$

where UV denotes use value, while Ui and N represent use reports and the number of informants, respectively.

#### Fidelity level

Some of the plants of one area are preferred in their utilization over others because of the effectiveness of the species. This preference is called fidelity level. The following formula is used to calculate the fidelity of the species [27].

5 $$FL\%=\frac{Ip}{Iu} \times 100$$

where *Ip* represents the proportion of informants who mentioned a species for a certain use. The number of informants who reported the same species for different uses is represented by the symbol *lu*. Fidelity levels vary from 1 to 100%. Values less than 100% show that the species is used for several purposes, while values closer to 100% show that it is utilized for a specific purpose.

#### Family importance value

The family importance value was calculated with the help of the subsequent formula. In the given formula ‘FIV’ represents the family importance value, while ‘RFC’ and ‘N’ denote the family frequency of citation of the family and the number of species within the family, respectively [27].

6 $$FIV=\frac{RFC}{N}$$

#### Jaccard distance

This index proves useful when the aim is to compare one community to another or when seeking to identify similarities and differences among two or more use categories. Its calculation involves the application of the following formula [28].

7 $$Jaccard\, Distance=1-Jaccard \,Index$$

## Results

**Fig. 2:**
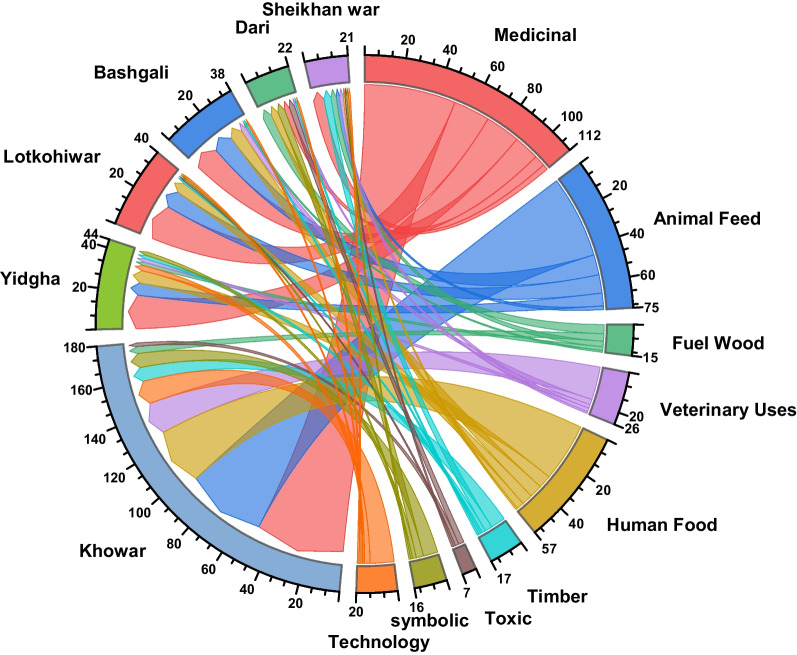
Chord diagram displaying affiliation of the linguistic group to the use categories

The research involved 120 native participants, consisting of 71 males and 49 females, representing various professional backgrounds. The respondents belonged to 6 linguistic backgrounds with age brackets ranging from 20 to over 60 years as displayed in Table 1. The objectives were to collect information on the extent, composition, function, and utilization of plant resources. The informants from diverse linguistic groups provided use reports about different plant groups as depicted by the chord diagram (Fig. 2). The participants identified 150 plant species belonging to 59 families encompassing 1 family of Pteridophytes, 3 gymnosperm families, and 55 angiosperm families.

### Use reports and informant consensus factor

In this work, use reports were systematically classified into nine principal categories, as outlined in Table 3. These categories served as representative groupings for the responses gathered across the entire study area. The distribution of use reports, along with the number of species associated with each use category, is presented in Table 4. The medicinal category displayed the highest species count, with 82 mentioned by respondents, yielding a total of 600 use reports. In contrast, the animal feed category encompassed 76 species, with a corresponding 500 use reports. The toxic use category comprised only 2 species, totaling 104 use reports. Furthermore, the informant consensus factor was markedly high, reaching 0.9 for all use categories, except for medicinal, animal feed, and human food categories, which displayed an informant consensus factor of 0.8.

**Table 4:** Consensus among informants regarding taxa usage across various use categories

Use category	N_taxa_	N_ur_	I_cf_
MED	82	600	0.8
AF	76	500	0.8
VU	21	450	0.9
TECH	26	320	0.9
HF	49	425	0.8
TIM	13	371	0.9
FW	12	400	0.9
SYM	8	320	0.9
TOX	2	104	0.9

### Relative frequency of citations

Table 5 shows the relative frequencies of taxa surveyed, highlighting their Relative Frequency of citations (RFC). *Platanus orientalis* and *Juglans regia* lead the list with an RFC of 0.91. Following closely are *Capparis spinosa*, *Morus alba*, *Thymus linearis*, and *Quercus baloot*, each boasting an RFC of 0.83. *Elaeagnus angustifolia* secures an RFC of 0.79. Additionally, *Cannabis sativa*, *Carum carvi*, and *Cucurbita maxima* exhibit significant presence, each reaching an RFC of 0.75.

**Table 5:** Quantitative assessment and utilization patterns of ethnobotanical taxa in Lotkuh, Chitral, Eastern Hindukush, Pakistan

Taxon/voucher number	Folk name	RFC	UR	FL%	UV	FIV	Use methods
**Pteridaceae** *Adiantum cappillus-veneris* L. Hafiz Bot. 01 [PUP]	Satjoshu	0.08	4	100	0.04	**0.08**	The powdered aerial parts are boiled and given to nursing mothers to enhance milk quantity
**Ephedraceae** *Ephedra gerardiana* Wall. ex Stapf Hafiz Bot. 02 [PUP]	Somani	0.41	20	75	0.20	**0.41**	The boiled extract is used as an anti-asthmatic. The ash after burning the leaves is mixed with fine tobacco to make wet snuff
**Cupressaceae** *Juniperus communis* L. Hafiz Bot. 03 [PUP]	Saruzjal	0.33	10	60	0.08	**0.42**	On the 21st of March, the Ismaili community burns it within their homes, creating smoke as a ritual to herald the commencement and ward off evil influences
*Juniperus excelsa* M. Bieb. Hafiz Bot. 04 [PUP]	Saruz	0.51	18	68	0.15		The gum from the stem is boiled in milk to deal with constipation. Shepherds cut the branches and dry them to use as off-season feed for cattle
**Pinaceae** *Pinus gerardiana* L. Hafiz Bot. 05 [PUP]	Jalghuza	0.66	20	62	0.23	**0.66**	The gum is boiled in water to create a thin syrup and used as an antispasmodic
**Juncaceae** *Juncus articulatus* L. Hafiz Bot. 06 [PUP]	Oghye	0.33	12	70	0.10	**0.29**	It is dried and fed to animals in winter as forage
*Juncus himalensis* Klotzsch Hafiz Bot. 07 [PUP]	Oghye	0.25	12	65	0.10		The green parts are boiled, and the extract is used against goat pox
**Poaceae** *Aristida cyanantha* Steud. Hafiz Bot. 08 [PUP]	Eshpur	0.41	10	100	0.50	**0.40**	Harvested in summer and is stored as feed for livestock in the winter season
*Phragmites karka* Retz. Hafiz Bot. 09 [PUP]	Gass	0.35	21	100	0.18		Serves as a premier feed option for donkeys and horses when mixed with silage
*Saccharum spontaneum* L. Hafiz Bot. 10 [PUP]	Noal	0.55	20	77	0.38		In spring the roots are taken out as nutritious feed for cattle by nomads. Nomads use it to construct a shelter roof with inclined angles as it does not retain
*Setaria italica* (L.) P. Beauv. Hafiz Bot. 11 [PUP]	Olen	0.27	20	66	0.46		Traditional bread, made from ground flour, is renowned for promoting stomach health. Millet wine is produced from seeds using traditional techniques
**Araceae** *Arum italicum* Mill Hafiz Bot. 12 [PUP]	Kolumikin	0.18	10	70	0.09	**0.18**	Birds like *Alectoris chukar* eat the seeds, while the leaves are fed to goats as medicine against a joint disease called the big knees
**Amaryllidaceae** *Allium carolinianum* DC. Hafiz Bot. 13 [PUP]	Jnglitestu	0.41	10	78	0.11	**0.50**	During summer, it is among the wild food plants collected and integrated into local cuisine. Some people consume it directly to alleviate dyspepsia
*Allium barszczewskii* Lipsky Hafiz Bot. 14 [PUP]	Latruk	0.33	10	78	0.15		The bulb is soaked in water and used to treat malaria. It serves human food as well as animal feed
*Allium chitralicum* F.T. Hafiz Bot. 15 [PUP]	Jnglitestu	0.50	16	78	0.13		The bulb is dried and eaten to manage hypertension. It is consumed as a nutritious wild food in fresh form
*Allium sativum* L. Hafiz Bot. 16 [PUP]	Wreznu	0.75	40	86	0.35		Boiled in water and the tea is taken to manage hypertension. It is a part of the daily cuisine
**Typhaceae** *Typha angustata* Bory & Chaub. Hafiz Bot. 17 [PUP]	Barwazi	0.18	12	100	0.10	**0.18**	The dried inflorescence is combined with sheep wool to craft cozy pillows
**Anacradiaceae** *Pistacia khinjuk* Stocks Hafiz Bot. 18 [PUP]	Binju	0.54	18	80	0.15	**0.54**	As a sacred plant, a piece of wood from its branch is crafted into an amulet to be worn around the neck of a newborn son, believed to protect against mortality
**Apiaceae** *Bunium persicum* Boiss. Hafiz Bot. 19 [PUP]	Hojuj	0.45	10	60	0.25	**0.45**	The bulb is used as raw snack, while the boiled seed extract is used to treat indigestion
*Carum carvi* L. Hafiz Bot. 20 [PUP]	Zeera	0.75	50	72	0.68		Ground seeds are used as a condiment and consumed with water to treat gastrointestinal disorders
*Ferula jaeschkeana* Vatke Hafiz Bot. 21 [PUP]	Ushye	0.64	40	78	0.50		The floral stalk with its epidermis removed is eaten with milk cream. Additionally, the dried stalk is boiled in milk to promote wound healing
*Foeniculum valgare* Mill Hafiz Bot. 22 [PUP]	Sonf	0.55	20	69	0.43		It serves as a condiment and its aqueous extract is utilized as an antipyretic
*Prangos pabularia* Lindl. Hafiz Bot. 23 [PUP]	Mushen	0.33	10	72	0.43		Primarily used as animal feed, the boiled extract is administered to individuals in cases of poisoning. It also serves as a feed to Markhor (*Capra falconeri*)
*Trachydium roylei* Lindl. Hafiz Bot. 24 [PUP]	Bngidwana	0.66	32	90	0.43		Used as feed in animal nutrition, consumption by sheep can lead to liver fluke attacks
**Apocynaceae** *Trachomitum venetum* L. Hafiz Bot. 25 [PUP]	Bakat	0.37	22	77	0.18	**0.37**	The plant fibers are spun using a spinning wheel to make ropes
**Asteraceae** *Anthemis cotula* L. Hafiz Bot. 26 [PUP]	Sherisht	0.66	40	87	0.46	**0.50**	The floral parts are boiled in water and administered to patients experiencing abdominal pain. They are also fed to cows to enhance the thickness of milk
*Artemisia brevifolia* Wall. ex DC Hafiz Bot. 27 [PUP]	Droon	0.41	25	66	0.60		Used as animal feed and fuel wood. The boiled extract is used as an anti-asthmatic
*Artemisia parviflora* Roxb. Hafiz Bot. 28 [PUP]	Karkalich	0.58	30	76	0.50		The plants are harvested and kept in houses as mosquito and insect repellent
*Cousinia chitralensis* Rech.f. Hafiz Bot. 29 [PUP]	Estorzukh	0.5	20	72	0.55		The floral parts are cooked and provided to pregnant women to promote vigor
*Tagetes minuta* L. Hafiz Bot. 30 [PUP]	Gulsambar	0.33	10	34	0.17		This plant holds deep significance in Khowar poetry, symbolizing the fragrance associated with the beloved
**Berberidaceae** *Berberis lycium* Royle Hafiz Bot. 31 [PUP]	Chuvenj	0.71	42	58	0.57	**0.60**	The fruits and the leaves are eaten directly as fresh food
*Berberis calliobotrys* Bien. Hafiz Bot. 32 [PUP]	Chuvenj	0.66	30	56	0.51		The roots are soaked in warm water and the extract is used to cure typhoid
*Berberis pseudumbellata* R. Parker Hafiz Bot. 33 [PUP]	Chuvenj	0.41	20	59	0.57		The fruits are collected and made a juice out of it and used as a liver tonic
**Betulaceae** *Betula utilis* D. Don Hafiz Bot. 34 [PUP]	Buli	0.58	25	70	0.36	**0.58**	Used as timber, the bark is soaked in water for purifying blood
**Boraginaceae** *Rochelia chitralensis* Y. Nasir Hafiz Bot. 35 [PUP]	Yorjoshu	0.25	9	68	0.07	**0.25**	The leaves are mortared and applied to a scorpion bite
**Brassicaceae** *Capsella bursa-pastoris* Medik. Hafiz Bot. 36 [PUP]	Zughjoshu	0.25	10	100	0.08	**0.37**	Collected as fodder for animals
*Nasturtium officinale* R. Br. Hafiz Bot. 37 [PUP]	Terghi	0.73	30	65	0.50		Directly eaten as food and as medicine against typhoid and malaria
*Lepidium sativum* L. Hafiz Bot. 38 [PUP]	Kardachi	0.26	10	100	0.25		Eaten directly and the oil from the seed is used to manage constipation
*Lepidium draba* L. Hafiz Bot. 39 [PUP]	Wahjoshu	0.25	12	88	0.18		It serves as animal forage, particularly for donkeys, as it is known to enhance donkey milk production
**Sapindaceae** *Acer caesium* Wall. Ex Brandis Hafiz Bot. 40 [PUP]	Dartelik	0.43	10	72	0.23	**0.43**	The wood is utilized for crafting polo sticks as well as general-purpose sticks
**Campanulaceae** *Codonopsis clematidea* C.B.Clarke Hafiz Bot. 41 [PUP]	Gundustak	0.33	25	68	0.27	**0.33**	The leaves are incorporated into a local recipe and cooked for consumption by lactating mothers
**Cannabaceae** *Cannabis sativa* L. Hafiz Bot. 42 [PUP]	Bong	0.75	45	100	0.62	**0.75**	Dried and fed to polo horses to keep their body warm during winter
**Rubiceae** *Galium chitralensis* Nazim. Hafiz Bot. 43 [PUP]	Birghal	0.36	10	100	0.29	**0.36**	Collected as a feed and is considered to increase the milk quantity in cattle
**Capparisdaceae** *Capparis spinosa* L. Hafiz Bot. 44 [PUP]	Kaveer	0.83	52	64	0.76	**0.83**	The floral buds are boiled in water and eaten as food and liver tonic
**Caryophyllaceae** *Silene conoidea* L. Hafiz Bot. 45 [PUP]	Apupar	0.41	6	100	0.09	**0.48**	It is collected from fields as fodder for livestock in early summer
*Vaccaria pyramidata* Medik. Hafiz Bot. 46 [PUP]	Satjoshu	0.55	5	78	0.06		Utilized both as feed and in veterinary treatment to enhance milk yield in cattle
**Amaranthaceae** *Beta vulgaris* L. Hafiz Bot. 47 [PUP]	Lablabu	0.66	30	54	0.36	**0.43**	Both roots and leaves serve as dietary staples. Roots are often stored in ditches during winter
*Chenopodium pamiricum* Iljin Hafiz Bot. 48 [PUP]	Sakh	0.58	12	62	0.18		Mountaineers in the wild commonly cook and consume the leaves
*Amaranthus viridis* L. Hafiz Bot. 49 [PUP]	Kharshakh	0.25	20	60	0.27		It is boiled in water and transformed into a local dish
*Chenopodium botrys* L. Hafiz Bot. 50 [PUP]	Shakh	0.43	13	65	0.25		During its early growth stages, it serves as human food, while in later stages used as animal feed
*Chenopodium foliosum* Asch. Hafiz Bot. 51 [PUP]	Pelilimrach	0.33	21	70	0.17		Humans consume the fruits directly, and they are also employed in treating hepatitis
*Haloxylon griffithii* (Moq.) Boiss. Hafiz Bot. 52 [PUP]	Pach	0.33	9	74	0.18		Cattles use it as fodder, while dried powder from leaves is applied to dry wounds
**Cucurbitaceae** *Cucurbita maxima* Duch.ex Lam. Hafiz Bot. 53 [PUP]	Aluk	0.75	30	88	0.66	**0.75**	Local dishes are prepared from pulp. The dried fruit shell serves as both a water container and a storage vessel for seeds
**Convolvulaceae** *Convolvulus arvensis* L. Hafiz Bot. 54 [PUP]	Pindormisk	0.5	10	100	0.16	**0.39**	It is collected from the field through weeding and used as fodder
*Cuscuta europaea* L. Hafiz Bot. 55 [PUP]	Umbool	0.25	10	98	0.18		Generally, as fodder, while soft yellow stem is collected to apply on pimples
*Cuscuta reflexa* Roxb. Hafiz Bot. 56 [PUP]	Umbool	0.43	20	98	0.17		The water obtained from the tissues is used to treat toothache
**Elaeagnaceae** *Elaeagnus angustifolia* L. Hafiz Bot. 57 [PUP]	Sinjur	0.79	62	89	0.81	**0.75**	The aroma of the beloved is described as akin to the fragrance emanating from this plant in poetry. Gums are used as hair tonic, while fruits are eaten directly
*Hippophae rhamnoides* L. Hafiz Bot. 58 [PUP]	Mirghinz	0.71	45	77	0.68		Commonly employed for fencing and as fuelwood. The berries are consumed directly to promote blood health, while they are also directly applied to the scalp as a skin moisturizer
**Fagaceae** *Quercus baloot* Griff. Hafiz Bot. 59 [PUP]	Banj	0.83	65	92	0.83	**0.83**	Wood is used for fuel. While leaves are used as feed for grazing animals
**Fumariaceae** *Fumaria indica* (Hausskn.) Linn. Hafiz Bot. 60 [PUP]	Shatara	0.25	20	100	0.16	**0.25**	Most widely used as animal feed
**Geraniaceae** *Geranium wallichianum* D. Don Hafiz Bot. 61 [PUP]	Rajuli	0.43	20	77	0.16	**0.38**	Within Khowar culture, the flowers of this plant bear symbolic significance, with the lips of the beloved being likened to the delicate petals of this flower
*Geranium parmiricum* Ikonn. Hafiz Bot. 62 [PUP]	Rajuli	0.33	11	100	0.09		The leaves are crushed, and a thick paste is prepared to eat for jaundice
**Grossulariaceae** *Ribes orientale* Desf. Hafiz Bot. 63 [PUP]	Sadabahar	0.33	5	100	0.04	**0.33**	This plant is widely used as veterinary medicine against liver flukes in cattle
**Plantaginaceae** *Hippuris vulgaris* L. Hafiz Bot. 64 [PUP]	Oghdronu	0.37	5	100	0.16	**0.37**	Mixed with silage to feed horses to treat equine influenza in horses
**Hypericaceae** *Hypericum perforatum* L. Hafiz Bot. 65 [PUP]	Matali	0.37	23	87	0.28	**0.37**	This plant holds cultural significance, as its name is frequently mentioned in Khowar songs as a form of praise for the beloved. Its petals are gathered and boiled in milk to treat throat infections
**Juglandaceae** *Juglans regia* L. Hafiz Bot. 66 [PUP]	Bermogh	0.91	50	60	0.82	**0.91**	The wood is crafted into utensils, while the oil is mixed with wheat flour to prepare a local pudding called Shoshp
**Lamiaceae** *Mentha longifolia* L. Hafiz Bot. 67 [PUP]	Ben	0.75	30	54	0.64	**0.70**	In early spring the fresh leaves are directly eaten as salad, while in later stages the inflorescence is dried and powdered and used with water against gas trouble
*Mentha spicata* L. Hafiz Bot. 68 [PUP]	Poodina	0.75	20	60	0.5		During early spring, the fresh leaves are consumed directly as a salad, while in later stages, the inflorescence is dried, powdered, and mixed with water to alleviate gas troubles
*Mentha royleana* Wall.ex Benth Hafiz Bot. 69 [PUP]	Ben	0.75	22	60	0.36		The dried vegetative parts are used as a constituent of local condiments
*Nepeta cataria* L. Hafiz Bot. 70 [PUP]	Mutrech	0.66	38	80	0.56		The dried floral buds are ingested with meals to aid in internal wound healing. In the wild, when Markhor sustains injuries, it consumes the herb for recovery
*Ocimum sanctum* L. Hafiz Bot. 71 [PUP]	Vorkrdchi	0.45	30	100	0.36		The leaves are added to local cuisines to improve the acidity of the stomach
*Otostegia limbata* Benth. ex Hook.f. Hafiz Bot. 72 [PUP]	Zehmuli	0.79	20	100	0.33		The boiled extract is given to sheep to treat the roundworm disease
*Salvia rhytidea* Benth. Hafiz Bot. 73 [PUP]	Sarjoshu	0.71	33	77	0.27		The powder derived from the leaves is added to dog bread to help keep them warm in winter
*Thymus linearis* Benth. Hafiz Bot. 74 [PUP]	Sew	0.83	28	63	0.54		A tea is made from it to treat heartburn and lower hypertension
*Ziziphora clinopodioides* Lam. Hafiz Bot. 75 [PUP]	Zughur	0.66	30	60	0.38		Tea is made from flowers to deal with intermittent flue. The lactating mother Markhor eats it profoundly
**Malvaceae** *Alcea rosea* L. Hafiz Bot. 76 [PUP]	Leen	0.66	16	70	0.22	**0.51**	A thick paste is prepared from the petal to treat tonsillitis
*Malva neglecta* Wallr. Hafiz Bot. 77 [PUP]	Yurpaghzu	0.36	20	68	0.17		Generally, as a feed for cattle. The leaves are eaten directly to deal with sore throat
**Moraceae** *Morus alba* L. Hafiz Bot. 78 [PUP]	Mrach	0.83	33	56	0.36	**0.75**	The wood is used to craft the Sitar, which is a major instrument in Chitrali folk music
*Morus nigra* L. Hafiz Bot. 79 [PUP]	Giltikan	0.66	20	52	0.34		The fruits are eaten directly as food and the juice is extracted to treat hepatitis and liver inflammation
**Oleaceae** *Fraxinus xanthoxyloides* Wall. Hafiz Bot. 80 [PUP]	Toor	0.64	23	56	0.45	**0.64**	The sturdy hardwood is devised into plows and essential agricultural implements
**Onagraceae** *Epilobium angustifolium* L. Hafiz Bot. 81 [PUP]	Bodoki	0.75	11	50	0.27	**0.75**	The flowers are collected and cooked to be eaten by nursing mothers
**Paeoniaceae** *Paeonia emodi* Royle Hafiz Bot. 82 [PUP]	Leenbash	0.75	12	100	0.10	**0.75**	The leaves are boiled, and the resulting infusion is filtered for drinking to combat intestinal worms
**Papaveraceae** *Papaver somniferum* L. Hafiz Bot. 83 [PUP]	Koknar	0.66	34	100	0.36	**0.66**	The fruit is pricked with blades, and the latex is subsequently dried to produce opium, which is used to alleviate body pain and chest infections
**Fabaceae** *Astragalus oihorensis* Ali Hafiz Bot. 84 [PUP]	Bespuk	0.45	20	67	0.25	**0.58**	It is fed to cattle to improve fertility in males and females
*Astragalus owirensis* Ali Hafiz Bot. 85 [PUP]	Bespuk	0.79	12	68	0.17		The leaves are boiled in water and the extract is taken against whooping cough
*Astragalus chitralensis* Ali Hafiz Bot. 86 [PUP]	Bespuk	0.71	30	54	0.34		Locals utilize it as firewood and provide it to their animals as fresh feed
*Astragalus gahiratensis* Ali Hafiz Bot. 87 [PUP]	Bespuk	0.83	10	50	0.11		It is used as animal feed
*Cicer microphylum* Royle Hafiz Bot. 88 [PUP]	Kuchun	0.66	23	60	0.19		It is dried in summer and used as fodder in winter
*Cicer nuristanicum* Kitam. Hafiz Bot. 89 [PUP]	Kuchun	0.66	20	62	0.16		The pods are boiled, and the filtrate is used as a potent antiarthritic
*Glycyrrhiza glabra* L. Hafiz Bot. 90 [PUP]	Moyo	0.62	26	77	0.35		It is employed to enhance male fertility and is utilized by individuals facing challenges in conceiving
*Lotus corniculatus* L. Hafiz Bot. 91 [PUP]	Zehch Josh	0.55	30	67	0.25		Cattles graze on it and is the most preferred species in alpine pastures
*Medicago minima* (L.) L. Hafiz Bot. 92 [PUP]	Agham	0.25	20	100	0.18		Harvested during the summer and stored for later use, often blended with wheat straw
*Medicago polymorpha* L. Hafiz Bot. 93 [PUP]	Agham	0.66	25	100	0.21		Harvested during the summer and stored for later use, often blended with wheat straw
*Melilotus officinalis* (L.) Lam. Hafiz Bot. 94 [PUP]	Agham	0.33	17	100	0.35		Harvested during the summer and stored for later use, often blended with wheat straw
*Sophora mollis* (Royle) Baker. Hafiz Bot. 95 [PUP]	Beshu	0.66	55	49	0.55		Locals harvest the plant to be used as fuel. It serves the purpose of fodder for browsers
*Oxytropis chitralensis* Ali Hafiz Bot. 96 [PUP]	Muser	0.37	21	70	0.50		Used as fodder
*Trifolium resupinatum* L. Hafiz Bot. 97 [PUP]	Shaftal	0.66	58	65	0.58		Used as fodder
*Vicia sativa* L. Hafiz Bot. 98 [PUP]		0.62	20	62	0.25		Poultry is provided with seeds to boost their egg-laying capacity
**Plantaginaceae** *Plantago lanceolata* L. Hafiz Bot. 99 [PUP]	Boeklegni	0.55	34	100	0.37	**0.40**	It is used both as human food and animal feed
*Plantago major* Aitch. Hafiz Bot. 100 [PUP]	Boeklegni	0.25	12	67	0.10		Used as animal feed
**Platanaceae** *Platanus orientalis* L. Hafiz Bot. 101 [PUP]	Chinar	0.91	44	52	0.36	**0.91**	A tree with symbolic importance, when within the bounds of a residence, is perceived as emblematic of elevated status and affluence in the community
**Polygonaceae** *Atraphaxis pyrifolia* Bunge Hafiz Bot. 102 [PUP]	Kanteli	0.33	20	100	0.16	**0.45**	The delicate branches are skillfully bound to fashion brooms
*Fallopia dumetorum* (L.) Holub. Hafiz Bot. 103 [PUP]	Polini	0.36	12	100	0.10		The plant is gathered after hand weeding and used as fodder
*Oxyria digyna* (L.) Hill Hafiz Bot. 104 [PUP]	Shutshakh	0.37	11	100	0.09		The leaves have been extensively used to treat Hepatitis C
*Persicaria maculosa* Gray Hafiz Bot. 105 [PUP]	Toqjoshu	0.26	11	100	0.09		Used as fodder for animals
*Rheum emodi* Wall. Hafiz Bot. 106 [PUP]	Ishpar	0.62	40	70	0.33		The floral stalk is eaten directly as one of the seasonal wild foods
*Rheum webbianum* Royle Hafiz Bot. 107 [PUP]	Ishpar	0.75	40	70	0.50		This plant can be consumed directly as food, while its juice is utilized for dissolving gallstones
*Rumex hastatus* D. Don. Hafiz Bot. 108 [PUP]	Shutshakh	0.33	20	68	0.50		It serves both as a direct food source and as a digestive tonic when consumed
*Rumex longifolius* DC. Hafiz Bot. 109 [PUP]	Serkonzu	0.66	14	100	0.53		The fresh leaves are picked and cooked with meat as local cuisine
*Cynanchum acutum* L. Hafiz Bot. 110 [PUP]	Ishperjosh	0.37	12	72	0.36		Collected from the wild as fodder for livestock
**Plumbaginaceae** *Acatholimon longiscapum* Bokhari Hafiz Bot. 111 [PUP]	Klabespuk	0.66	30	68	0.26	**0.49**	Because of its cushion-like structure, shepherds in the wild use it for straining milk and feed as well
*Psillostachys suworowii* Roshkova Hafiz Bot. 112 [PUP]	Gulandam	0.33	13	100	0.23		The leaves are collected as fodder for cattle
**Portulacaceae** *Portulaca oleracea* L. Hafiz Bot. 113 [PUP]	Pechili	0.75	20	100	0.33	**0.75**	Cooked with other vegetables as a part of daily diet
**Primulaceae** *Primula denticulata* Smith. Hafiz Bot. 114 [PUP]	Punar	0.50	26	77	0.33	**0.50**	Dew drops gathered on the corolla tube are introduced into the eyes to alleviate pink eye disease and symbolic
**Punicaceae** *Punica granatum* L. Hafiz Bot. 115 [PUP]	Dalum	0.25	36	59	0.45	**0.25**	The dried rinds are powdered and taken with water to address stomach ulcers
**Ranunculaceae** *Clematis graveolens* Lindl. Hafiz Bot. 116 [PUP]	Chontruk	0.43	32	56	0.33	**0.44**	The leaves are pressed to extract water, which is then applied to combat ringworm infections
*Clematis orientalis* L. Hafiz Bot. 117 [PUP]	Chontruk	0.33	30	67	0.31		Used as fodder, while the leaves are incorporated into poultry feed to mitigate flu outbreaks
*Ranunculus muricatus* L. Hafiz Bot. 118 [PUP]	Wahjoshu	0.66	12	87	0.10		Used as fodder as well as veterinary medicine to treat diarrhea in calves
*Ranunculus natans* C.A. Mey. Hafiz Bot. 119 [PUP]	Wahjoshu	0.37	10	68	0.08		As veterinary medicine to treat diarrhea in calves
**Rosaceae** *Sibbaldia procumbens* M. Bieb. Hafiz Bot. 120 [PUP]	Bronjoshu	0.66	10	100	0.43	**0.62**	Used as fodder for animals in the wild
*Cotoneaster affinis* Lindl. Hafiz Bot. 121 [PUP]	Bechoshi	0.33	20	63	0.16		Fruits are eaten and the powdered seeds are utilized to eliminate kidney stones
*Malus domestica* (Suckow) Borkh. Hafiz Bot. 122 [PUP]	Boop	0.75	30	70	0.41		The dried fruits are powdered and ingested with water to relieve chest infections
*Prunus prostrata* Labill. Hafiz Bot. 123 [PUP]	Meken	0.75	21	70	0.17		The fruits are eaten by the Himalayan snowcock (*Tetraogallus himalayensis*)
*Crataegus songarica* K. Koch. Hafiz Bot. 124 [PUP]	Guni	0.50	25	70	0.21		The fruits serve as food as well as cardiotonic, while the wood is used in crafting polo balls
*Prunus amygdalus* Batsch Hafiz Bot. 125 [PUP]	Badam	0.66	24	55	0.20		In the wild, the leaf of wild almond is the preferred diet of the markhor
*Prunus armeniaca* L. Hafiz Bot. 126 [PUP]	Zuli	0.75	40	100	0.67		The sun-dried fruits are sold in the market as a source of income for locals
*Prunus domestica* L. Hafiz Bot. 127 [PUP]	Alucha	0.58	30	100	0.58		Used as edible fruits
*Prunus persica* (L.) Bastch Hafiz Bot. 128 [PUP]	Girgalokh	0.66	44	100	0.64		Used as edible fruits
*Pyrus communis* L. Hafiz Bot. 129 [PUP]	Shoghori	0.73	62	100	0.67		Used as edible fruits
*Prunus avium* L. Hafiz Bot. 130 [PUP]	Gilas	0.58	31	67	0.55		Local jams and jellies are made from it
*Rosa alba* L. Hafiz Bot. 131 [PUP]	Gulab	0.50	30	77	0.35		The petals are used to make tea
*Rosa brunonii* Lindl. Hafiz Bot. 132 [PUP]	Gulab	0.66	24	54	0.20		The petals are boiled to make tea
*Rosa webbiana* Wall. ex Royle Hafiz Bot. 133 [PUP]	Thorni	0.75	26	52	0.35		The petals are boiled to drink for throat infection. The fruits are eaten by the doves
**Saxifragaceae** *Bergenia ciliata* Sternb. Hafiz Bot. 134 [PUP]	Asqarbash	0.58	20	100	0.28	**0.58**	The leaves are fried and eaten to increase milk production in nursing mothers
**Salicaceae** *Populus alba* L. Hafiz Bot. 135 [PUP]	Romenu	0.66	32	50	0.35	**0.63**	Used to make furniture and other house structures
*Populus nigra* L. Hafiz Bot. 136 [PUP]	Terek	0.73	30	52	0.25		Used as major timber for building rafters
*Populus euphratica* Olivier Hafiz Bot. 137 [PUP]	Terek	0.58	14	56	0.51		Used to make doors, beams, and other building structures
*Salix acmophylla* Boiss. Hafiz Bot. 138 [PUP]	Chikar	0.5	33	49	0.27		The elastic branches are used to make baskets to carry luggage
*Salix alba* L. Hafiz Bot. 139 [PUP]	Teli	0.66	48	48	0.55		Inflorescences are boiled to create a paste applied on newborns' faces to protect them from cold and sunburn
*Salix iliensis* Regel Hafiz Bot. 140 [PUP]	Teli	0.75	30	51	0.43		Used as animal feed and fencing around kitchen gardens
*Salix tetrasperma* Roxb. Hafiz Bot. 141 [PUP]	Teli	0.58	32	60	0.26		Used as animal feed in early spring when stored feed of the winter is exhausted
**Scrophulariaceae** *Verbascum thapsus* L. Hafiz Bot. 142 [PUP]	Gurdoghkaru	0.66	27	100	0.45	**0.66**	The leaves are simmered and administered to those suffering from epilepsy
**Solanaceae** *Datura stramonium* L. Hafiz Bot. 143 [PUP]	Zaqum	0.73	22	100	0.51	**0.68**	Called toxic and are recommended to avoid
*Hyoscyamus niger* L. Hafiz Bot. 144 [PUP]	Bangdewana	0.58	14	100	0.35		Called toxic and are recommended to avoid
*Solanum nigrum* L. Hafiz Bot. 145 [PUP]	Pirmilik	0.73	5	100	0.28		Fruit is used as an ointment and applied on the scalp to remove pimples
**Tamaricaceae** *Myricaria elegans* Royle Hafiz Bot. 146 [PUP]	Papaki	0.58	10	100	0.36	**0.50**	Locals use it for fencing around kitchen gardens
*Tamarix dioica* Roxb.ex Roth. Hafiz Bot. 147 [PUP]	Hinju	0.5	5	65	0.35		Polo sticks are made from wood as it is delicate and does not break easily
**Violaceae** *Viola serpens* Wall. ex Ging. Hafiz Bot. 148 [PUP]	Mulkon	0.66	19	58	0.36	**0.66**	Used as food and the leaves are added to local dishes
**Nitrariaceae** *Peganum harmala* L. Hafiz Bot. 149 [PUP]	Ispandur	0.75	30	100	0.35	**0.75**	It is smoldered in homes to remove the effect of evil eyes
**Zygophyllaceae** *Tribulus terrestris* Linn. Hafiz Bot. 150 [PUP]	Meshenji	0.58	2	100	0.38	**0.58**	The powder is combined with the bread of hunting dogs to prevent them from feeling cold

Bold represent the family importance value

RFC = Relative Frequency of citation; UR = Use report; FL = Fidelity Level; UV = Use Value; FIV = Family Importance Value

### Use value

**Fig. 3:**
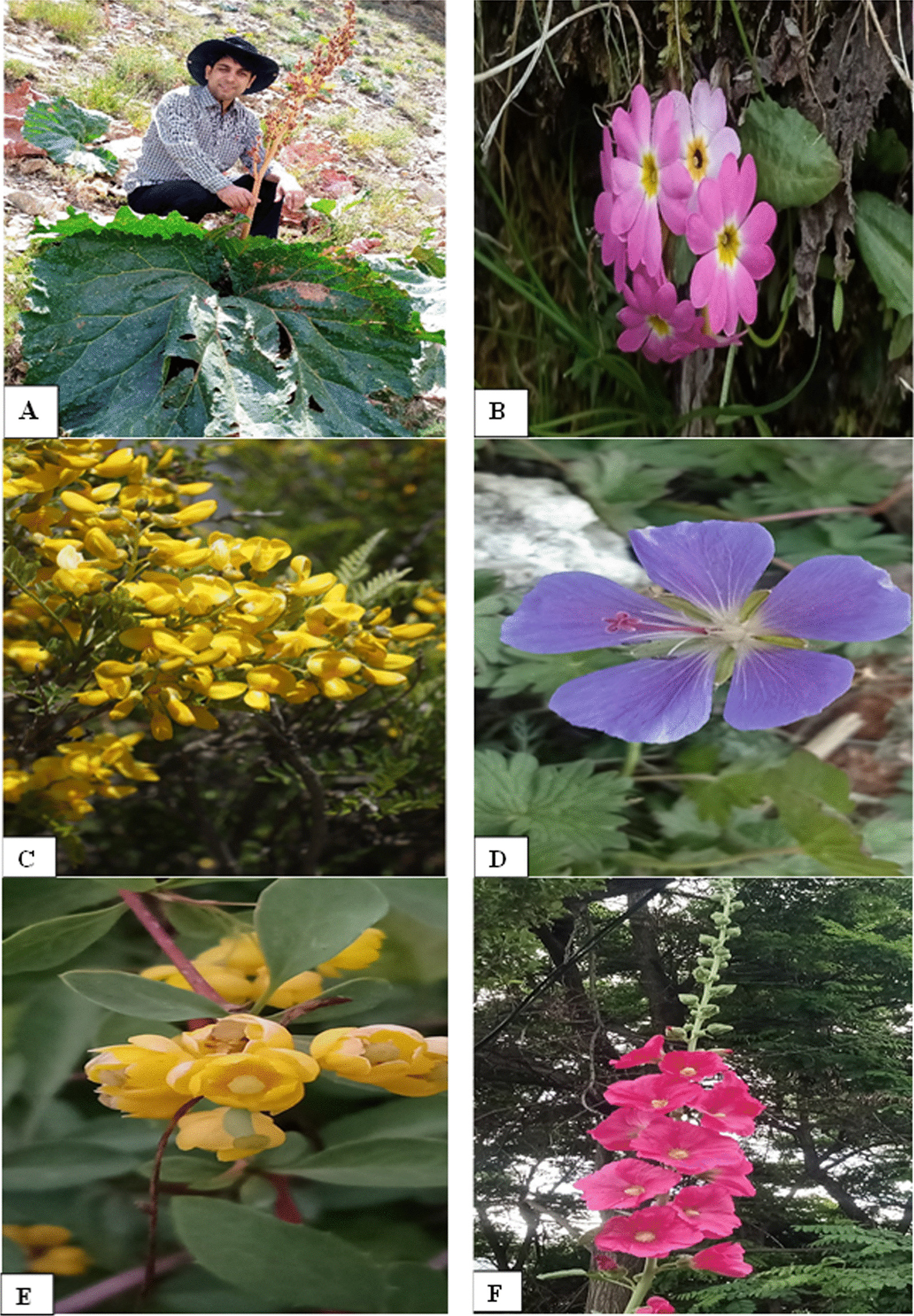

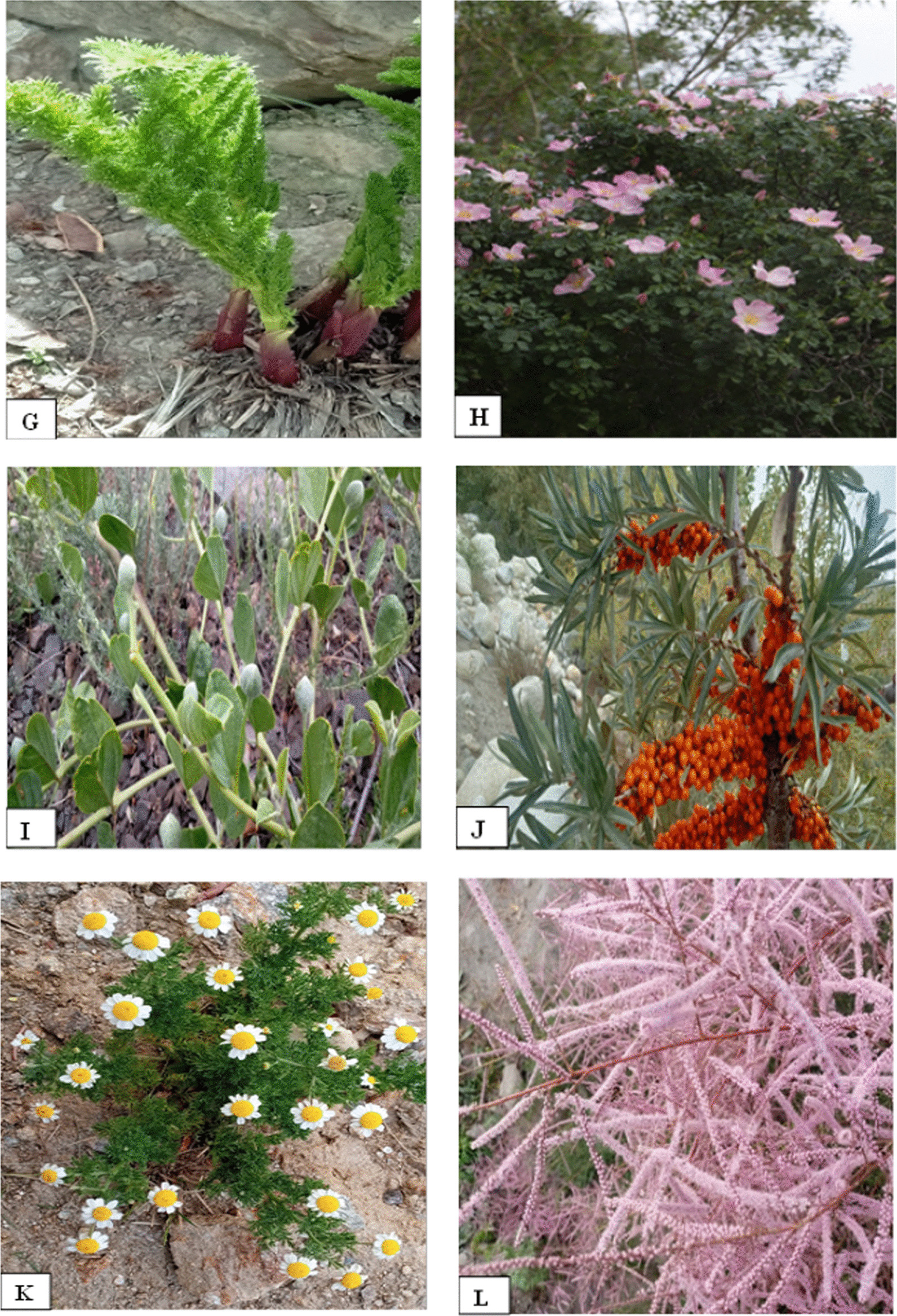
Some of the ethnobotanically quoted plants. **A** *Rehum webbianu*m **B** *Primula denticulata* **C** *Sophora mollis* **D** *Geranium wallichianum* **E** *Berberis lyceum* **F** *Alcea rosea* **G** *Prangos pabularia* **H** *Rosa webbiana* **I** *Capparis spinosa* **J** *Hiphophae rhamnoides* **K** *Anthemis cotula* **L** *Tamarix dioica*

The use values (UV) of the plant species surveyed are presented in Table 5. Conspicuously, *Juglans regia* stands out with a UV of 0.82, followed closely by *Elaeagnus angustifolia* at 0.81, and *Capparis spinosa* at 0.76. Other significant species include *Rheum webbianum* with a UV of 0.75, Carum *carvi* 0.68, *Ferula jaeschkeana* 0.50, *Cucurbita maxima* at 0.66, *Hippophae rhamnoides* at 0.68, *Berberis lyceum* at 0.57, and *Nasturtium officinale* a UV of 0.50. Some of the plants with higher use values are shown in Fig. 3. Use values provide an insight into the relative importance of each species in the context of their utilization, a necessary aspect of our research findings.

### Fidelity level

Table 5 presents the fidelity levels of the taxa considered in the study, comprising a total of 42 species with a 100% fidelity level. Prominent examples of these highly faithful species include *Adiantum cappillus-veneris*, *Aristida cyanantha*, *Peganum harmala,* etc. Conversely, the lowest fidelity level, at 34%, was observed in *Tagetes minuta*. The plant species exhibiting 100% fidelity level span various utilization categories, such as medicinal, human food, animal feed, technology, and toxic. Specifically, the animal feed category displays the highest number of plants with 18 species at 100% fidelity, followed by 13 species in the medicinal category. Human food encompasses 7 species with perfect fidelity, while both the toxic and technology categories feature 2 species each. Amidst the spectrum of fidelity levels, there are species like *Cuscuta europaea* (98%), *Trachydium roylei* (90%), and *Quercus baloot* (92%).

### Family importance value

In Table 5, we present the family importance values (FIV) of the surveyed families, showcasing a remarkable diversity in their significance. Markedly, Juglandaceae and Platanaceae emerge as the top-ranking families, each boasting a high FIV of 0.91. Following closely, Capparidaceae achieves a notable FIV of 0.83, while Paeoniaceae, Nitrariaceae, Portulacaceae, and Cannabaceae also contribute significantly with FIVs ranging around 0.75.

These elevated FIVs for specific plant families underscore their pronounced importance within the cultural context. On the other hand, certain families exhibit lower FIVs, indicating comparatively lesser cultural significance. Notably, Boraginaceae, Typhaceae, Araceae, and Pteridaceae secure lower FIVs, ranging from 0.08 to 0.25. The contrasting FIV values shed light on the varying degrees of cultural importance attributed to different plant families within the study area.

### Jaccard distance

**Fig. 4:**
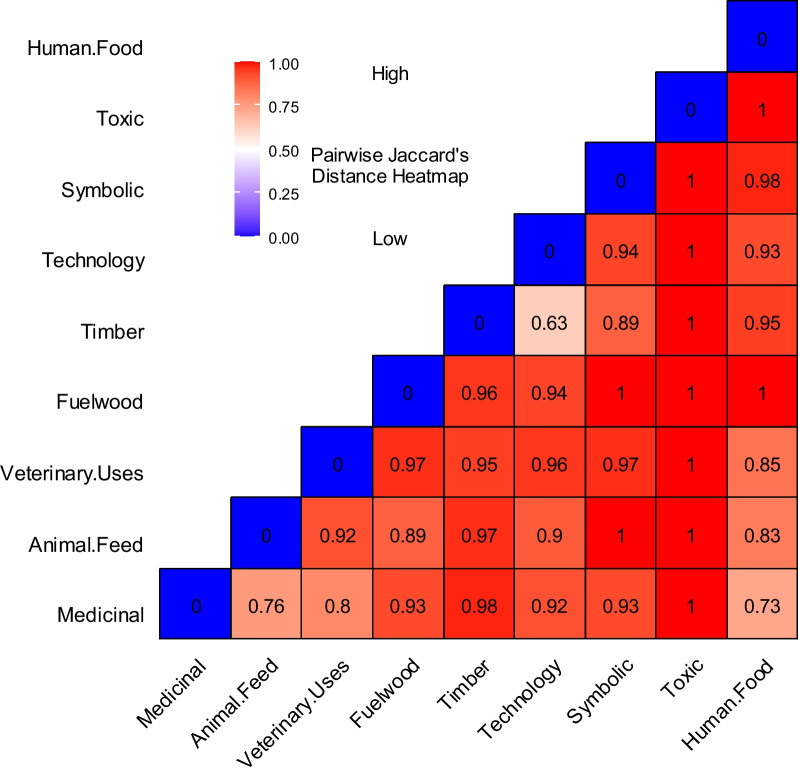
Pairwise Jaccard’s distance heatmap showing the distance between the use categories

Jaccard distance is displayed in Fig. 4. It varies between 0.63 as the minimum and the highest value of 1. The use category (Toxic) in at maximum Jaccard distance with all other groups showing zero similarity with the rest of the use groups, while the least distance (0.63) was observed between (the Timber and Technology) use groups. The Jaccard distance heat map dendrogram shows the clustering of the use categories. As the species in different use groups overlap, the dendrogram has three major clusters at the base.

## Discussion

### A comparative analysis of data with existing literature in Pakistan

A comparative analysis was conducted on the types and utilization of ethnobotanically significant taxa, juxtaposed with existing data, to discern unique plant species and distinct cultural uses prevalent in the study area. The study presents new ethnobotanical species and approaches for utilization that have not previously been documented. The medicinal category stands out with the highest number of use reports, totaling 600 as detailed in Table 4. This keen reliance on medicinal plant use can be attributed to the challenging geographical terrain of the Hindukush region and the lack of essential healthcare facilities, as highlighted in a prior study [29]. Though literature is available on the medicinal plants of northern Pakistan [30], we report some novel species, novel methods, and target uses. *Astragalus oihorensis* is used to improve infertility in males and females, while *Astragalus owirensis* is used against whooping cough. Similarly, *Cicer nuristanicum, Geranium parmiricum, Rochelia chitralensis,* and *Cousinia chitralensis* are new species with traditional medicinal uses reported in this work. *Adiantum cappillus-veneris* is widely reported from many parts of northern Pakistan [6, 31], but it is used to deal with human infertility in this region. *Ephedra gerardiana* is widely mentioned with medicinal uses [6, 32], while in this region it is widely used to make snuff and sold in the market, a threat to slowing growing and less abundant species [33]. *Nepeta cataria* is the herb consumed by Markhor, soon after the animal sustains injuries, thus a wild healing agent [34]. *Primula denticulata* has been used for disorders like asthma, and bronchitis [35], to treat cattle disease [36], but not for pink eye disorder as our study suggests.

The animal feed category encompasses 76 species and 500 use reports. Livestock rearing serves as the primary means of sustenance for those residing in the mountainous regions, a reality consistent with the inhabitants of the study area located in the eastern Hindukush [6]. The plants in the wild of the study area are not only sources of domestic animals but also sustain the life of wild ungulates like the Markhor (*Capra falconeri cashmeriensis*) which relies on *Nepeta cataria* as medicine when wounded and forages *Ziziphora clinopodiodes* during lactation. Such plant species are the backbone to sustain the conservation of iconic species like the Markhor [37]. The Himalayan snow cock (*Tetraogallus himalayensis*) eats *Prunus prostrata* and the Chukar partridge (*Alectoris chukar*) consumes the fruit of *Arum italicum.* Conservation of the unique wildlife of this area is directly associated with some of the key plant species [38].

Livestock rearing and herding are integral to the livelihoods of the people in this area, serving as the cornerstone of their existence. Certain plant species such as *Quercus baloot*, *Juniperus excelsa,* and *Betula utilis* exhibit sparse populations and slow growth rates, making them particularly vulnerable to significant consumption pressures. These species were frequently cited, highlighting the intensity of the demands placed upon them [39].

Both urban and rural communities have long prioritized the health of their domesticated and companion animals. In contrast to urban people, residents of the mountains choose wild plants for the good health of their animals [40]. Given the limited access to human healthcare facilities in this area, the challenge is exacerbated when it comes to addressing animal health. Nomads, shepherds, and pastoralists have traditionally relied on plant-based remedies for the well-being of their animals since history and the practice continues [41]. *Cannabis sativa* was used as feed for polo horses to keep them warm in winter, while in most literature it is a potent drug [42]. In this cultural context, the utilization of *Juncus himalensis* for treating goat pox, *Trachydium roylei* for combating liver fluke attacks, *Hippuris vulgaris* for addressing equine influenza in horses, *Astragalus oihorensis* for enhancing cattle fertility, *Tribulus terrestris* for maintaining warmth in hunting dogs, are not visible within ethnobotanical literature [43–45].

The human food category has 425 use reports by the informants and embraces 49 species. Our research has shed light on unconventional food plants within the region, with a focus on enhancing food security. *Allium chitralicum* and *Allium carolinianum* are among the wild food plants remembered by respondents for their historical uses, particularly during times of famine [2, 44]. *Pinus gerardiana, Ferula jaeschkeana, and Rheum webbianum* not only serve as edible resources but also constitute the primary sources of income for the local population. However, the methods and quantities in which these resources are collected currently contradict sustainable practices. It is imperative to provide the local community with training on the judicious utilization of WFPs [33]. *Capparis spinosa, Chenopodium pamiricum, Chenopodium foliosum, and Thymus linearis* are also common wild food plants. However, the collection of floral buds of *Capparis spinosa* is widespread, discouraging seed production [46]. Many informants have recognized edible wild plants that contain ample nutritional and mineral content capable of meeting human dietary requirements [47].

Within the culturally vibrant study region, plants hold significance not only for their utilitarian purposes but also for their aesthetic appeal and role in cultural celebrations[48]. Certain plants are deeply intertwined with the local belief systems, adding layers of meaning and symbolism to their use [49]. Wearing a wooden amulet made from *Pistacia khibenjuk* around the neck is thought to offer protection to newborns, shielding them from mortality. On the 21st of March, *Juniperus communis* is smoked inside households as part of a religious observance to mark the start of the new year. This ritual is believed to serve as a safeguard against malevolent influences and illnesses throughout the upcoming year [50].

In the realm of Khowar poetry, verses sway with the grace of *Elaeagnus angustifolia*, *Tagetes minuta*, and *Primula denticulata*. Poets, fascinated by the fragrance and colors of these plants, weave them into metaphors to extol the beauty of their beloveds. These plants hold unique symbolism within Khowar poetry, not referenced in other works [51]. The data elucidated several culturally significant crafts and tools unique to the local community, distinguishing them from practices found in other cultures. *Trachomitum venetum* is used to weave ropes, while *Acer caesium* serves as the primary material for crafting polo sticks [52]. *Fraxinus xanthoxyloides* finds its purpose in the creation of agricultural implements. The Chitrali sitar, a long-necked, plucked-string musical instrument, is crafted from the wood of *Morus alba* [50]. These plants not only carry cultural significance but also contribute to the local economy through cash earnings and offer potential for cultivation and sustainable utilization.

Table 4 indicates that 13 taxa serve as timber sources, while 12 are utilized for fuelwood purposes. Among the most prevalent timber species identified are *Populus alba, Populus nigra, Salix alba, Morus alba, Platanus orientalis, Morus nigra, Betula utilis,* and *Juniperus excelsa*. Of specific concern are *Betula utilis* and *Juniperus excelsa*, which face extensive utilization and have few remaining patches of vegetation [53].

In terms of fuelwood, *Quercus baloot, Juniperus excelsa, Artemisia brevifolia*, and *Sophora mollis* emerge as the most frequently cited species. The data highlight the necessity for providing alternative energy options to residents to lighten the consumption pressure on these species [54]. The use category labeled as 'toxic' exhibits the least number of species, with 104 use reports. The residents of this region possess knowledge about the potentially harmful flora and actively discourage the utilization of such species by humans [55].

### Informant consensus factor

Table 3 presents the informant consensus factor (ICF) for the nine identified use categories in this study. Overall, the ICF ranges from 0.8 to 0.9, indicating a relatively high level of agreement among informants. Categories such as TECH, VU, TIM, FW, SYM, and TOX exhibit an ICF of 0.9, while MED, AF, and HF have an ICF of 0.8. The ICF reflects the consensus among informants regarding the specific uses of plant species, with values ranging from 0 to 1. Values closer to 1 suggest greater agreement among respondents on taxon utilization, while values closer to 0 indicate either disagreement, species diversity, or less shared information among informants [56]. Our results indicate a substantial level of agreement among informants, suggesting shared knowledge about the benefits of plants for specific purposes. This trend is principally marked in rural areas within mountain landscapes, where limited urbanization exists, and residents heavily rely on plant resources.

### Relative frequency of citation

The significance of a taxon to a specific culture is determined by its relative frequency of citation. Well-known species tend to be the most utilized [57]. *Platanus orientalis* and *Juglans regia* have exceptionally high relative frequencies of citations, both attaining 0.91. As illustrated in Table 5, these species are versatile, contributing to various use categories such as technology and craft (TECH), timber (TIM), symbolic (SYM), and human food (HF). Their diverse roles make them vital to the local community, establishing their regional importance [25, 58]. Following closely are *Capparis spinosa*, *Morus alba*, *Thymus linearis*, and *Quercus baloot*, each boasting an RFC of 0.83. *Elaeagnus angustifolia* secures an RFC of 0.79. Additionally, *Cannabis sativa*, *Carum carvi*, and *Cucurbita maxima* exhibit significant presence, each reaching an RFC of 0.75. Plants with economic significance tend to capture the interest of local communities, a trend observed in many Asian countries [59].

### Fidelity level

Table 5 presents the fidelity levels of the taxa considered in the study, comprising a total of 42 species with a fidelity level of 100%. Prominent examples of these highly faithful species include *Adiantum cappillus-veneris*, *Aristida cyanantha*, *Peganum harmala,* etc. Certain species flash more in one specific use category compared to others, and informants express greater confidence when revealing such a taxon [60]. Specifically, the animal feed category displays the highest number of plants with 18 species at 100% fidelity. The snow-capped mountains provide seasonal fodder for animals, and herders are required to select the best options for their livestock [61, 62]. In the medicinal category, 13 species show 100% fidelity showing that people have assigned specific roles to some plants specifically curing ailments [63]. Human food encompasses 7 species with perfect fidelity indicating the knowledge of the people about the nutritious plants in the mountains [64, 65]. Amidst the spectrum of fidelity levels, there are species like *Cuscuta europaea* (98%), *Trachydium roylei* (90%), and *Quercus baloot* (92%), reflecting a historical and enduring use by the community.

### Family importance value

Table 5 demonstrates the family importance values (FIV) of the families surveyed, revealing a diversity in their significance. Markedly, Juglandaceae and Platanaceae stand out as the top-ranking families, each boasting a substantial FIV of 0.91. The significance of Juglandaceae, exemplified by species like *Juglans regia*, extends to the local communities due to its multifaceted contributions in terms of nutrition, medicine, and income generation [64, 65]. Platanaceae, particularly with the presence of *Platanus orientalis* as a primary source of timber production, holds greater importance. Furthermore, the family carries a significant symbolic value, with the presence of *Platanus orientalis* in the yard being regarded as a mark of distinction for a noble family [66]. Following closely, Capparidaceae achieves an FIV of 0.83 as it is a source of medicinal and food plants. Capparis spinosa is the widely consumed herb in the area in local cuisine and a broad-spectrum medicinal plant as well [67]. High FIVs for specific plant families underscore their pronounced importance within the cultural context. On the other hand, certain families exhibit lower FIVs, indicating comparatively lesser cultural significance. Notably, Boraginaceae, Typhaceae, Araceae, and Pteridaceae secure lower FIVs, ranging from 0.08 to 0.25.

### Jaccard distance

**Fig. 5:**
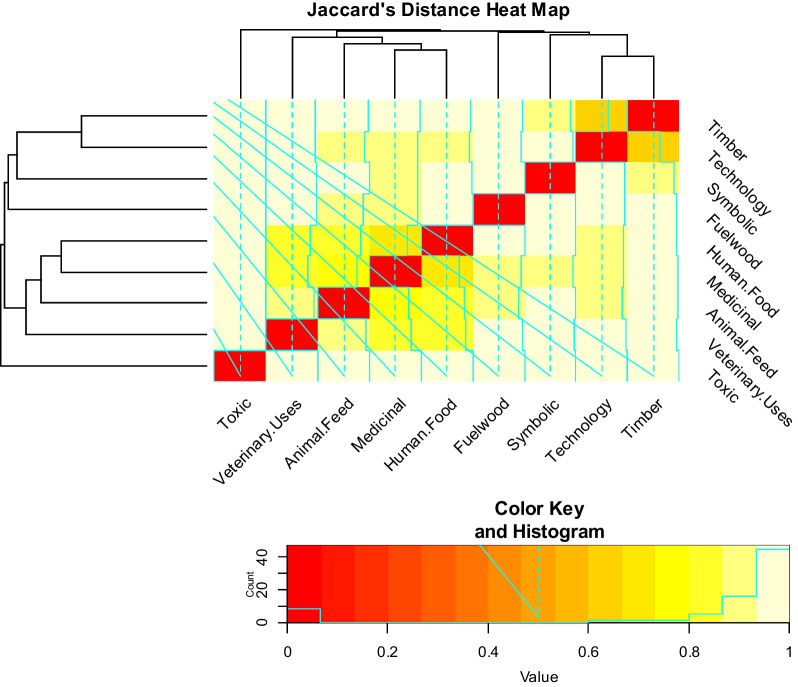
Jaccard distance heatmap dendrogram showing the cluster of use categories

The Jaccard distance, depicted in Fig. 4, and a cluster dendrogram in Fig. 5 show range from 0.63 to its highest at 1. Certain taxa establish versatility with numerous use reports, resulting in lower Jaccard distances, whereas other species exhibit fidelity to a single-use group, leading to maximum distances. Conspicuously, the use category "Toxic" exhibits the maximum Jaccard distance with all other groups, indicating zero similarity with the rest of the use groups. Conversely, the smallest distance (0.63) is observed between the "Timber" and "Technology" use groups as both share tree species [4].

### Data novelty in terms of food security, public health, and environment

The findings of this study shed light on the intricate relationship between environmental changes and food security, particularly in the Hindukush region of northwestern Pakistan. As climate change exacerbates, communities across this area, characterized by their reliance on mountain ecosystems, face amplified vulnerability [68]. With the looming risks to food security in the Hindukush Himalayas, the mountain dwellers are expected to encounter persistent insecurity challenges in the future [69]. We discovered that a variety of wild plants were frequently incorporated into both traditional cuisines and consumed raw as snacks. *Allium carolinianum, Allium barszczewskii, Allium chitralicum, Cotoneaster affinis, Prunus prostrata, Rheum emodi, Rheum webbianum, Crataegus songarica, Elaeagnus angustifolia, Berberis lyceum, Ferula jaeschkeana, Morus nigra, Mentha royleana,* and *Mentha longifolia* are some of the most mentioned raw snakes shown in Table 5. This reliance on wild food plants is not merely a historical practice but rather an ongoing aspect of daily sustenance, indicating a profound adaptation of local communities to their environment [70]. The study highlights the mobility of pastoralists and common locals who traverse the mountains, particularly for seasonal harvests of these wild food plants [71]. Thus, the insights garnered from this study offer valuable perspectives on the multifaceted dimensions of food security within the context of environmental change and local adaptation strategies. 

In addition to raw snakes, the local community harvests a variety of wild vegetables with high nutritional value, which are then freshly prepared in diverse local cuisines [72] or preserved for winter consumption, ensuring a year-round food supply. *Allium carolinianum, Nasturtium officinale, Codonopsis clematidea, Capparis spinosa, Amaranthus viridis, Plantago lanceolata, Rumex longifolius, and Portulaca oleracea* are commonly cited by locals as staple ingredients in their cuisine [41]. These species have historically served as vital sources of sustenance during periods of famine and political turmoil, offering nourishment even across borders during conflicts [73]. Therefore, it provides understanding into the species that could potentially serve as food when necessary.

Public health is a major concern in mountains of northern Pakistan [74]. This research sheds light on previously undocumented plants in Pakistani ethnobotanical literature, as well as innovative uses for familiar species. Among the noteworthy discoveries are *Astragalus owirensis*, *Cicer nuristanicum*, *Geranium parmiricum, Rochelia chitralensis*, and *Cousinia chitralensis*, which emerge as frequently cited novel species. According to an estimate out the 6000 medicinal plant species present in Pakistan 700 are medicinally important [75]. The novel species documented in the current ethnobotanical study have the potential to integrate into folk medicine, aligning with the World Health Organization's advocacy for alternative medicines and preventative healthcare, especially in developing nations [76]. In Pakistan, the traditional Unani and Ayurvedic healing systems are gaining recognition for their therapeutic efficacy. However, the traditional knowledge associated with valuable medicinal plants faces significant threats to its preservation. In Pakistan the Unani and Ayurvedic systems have been increasing therapeutic qualities, but the valuable medicinal plants knowledge system is threatened [77].

In addition to identifying novel species, this study also explores innovative uses for familiar ones. For instance, the utilization of *Adiantum cappillus-veneris*, as documented by in addressing human infertility, is a noteworthy discovery [6, 31]. *Ephedra gerardiana* is being processed into snuff and commercially traded, posing a threat to its sustainability and the conservation of less common species. *Nepeta cataria*, recognized traditionally (Kakakhel, 2020), has been observed in this study as a wild healing agent for Markhor, the iconic national animal of Pakistan. Similarly, while *Primula denticulata* has historically been employed for treating ailments such as asthma and bronchitis [35] and even utilized in veterinary medicine for cattle diseases [36], our research uncovers its potential efficacy in treating pink eye disorder, expanding its known applications.

Ethnobotany offers profound insights into the myriad of plant species and their sustainable utilization to address pressing environmental issues [78]. In our research area, the sparse vegetation is a result of its location within the dry temperate rain shadow zone. Our findings reveal that certain plant species serve multifaceted roles and are frequently cited. However, these species face vulnerability due to increased consumption. For instance, *Quercus baloot* is noted by informants as a primary source of fuelwood, yet it also serves as the sole winter forage for wild herbivores like the Markhor when snow blankets the landscape [37]. *Rheum webbianum*, a scattered species, is a highly demanded wild food plant, while the collectors uproot it in during harvest, a method that can question the conservation of the species [79]. *Artemisia brevifolia* as seen in the field study is the major soil binder, while it has a relatively high use value of 0.60 and is uprooted to collect for fuel. Such a practice has already caused flash floods in the study area [80]. Some rare gymnosperms like *Ephedra gerardiana* with a high frequency of citation 0.41 and fidelity level of 75 are burnt to ash to make snuff that is sold in the market. Again, such a process can cause environmental degradation as the species density is comparatively low in the region.

## Conclusion

The study identified 150 plant species across 59 families, unveiling novel ethnobotanical species, methods, and purposes of usage. For instance, *Astragalus oihorensis* is used to improve human infertility, while *Astragalus owirensis* for treating whooping cough. Similarly, *Cicer nuristanicum, Geranium parmiricum, Rochelia chitralensis,* and *Cousinia chitralensis* are novel species with traditional medicinal uses. The use of *Adiantum cappillus-veneris* for human infertility and *Ephedra gerardiana* in commercial-scale snuff are novel uses reported. The use of *Nepeta cataria,* by Markhor shortly following injuries, is new to ethnobotany*.* Previous studies show *Primula denticulata* is effective against asthma and bronchial issues. Our study suggests it to treat pink eye disorder. The medicinal category with 600 reports, implies the local population's exclusive dependence on medicinal plants. The ‘toxic’ category with 104 reports, denotes the community’s awareness of plants harmful to humans. *Platanus orientalis* and *Juglans regia* have high (0.91) citation frequencies, followed closely by *Capparis spinosa, Morus alba, Thymus linearis,* and *Quercus baloot* at 0.83. The animal feed category encompasses 18 species with 100% fidelity, indicating their special role as feed. Similarly, in the medicinal category, 13 species exhibit 100% fidelity, highlighting them as exceptional local medicine. In family rankings, Juglandaceae and Platanaceae secure top positions with a substantial family importance value (FIV) of 0.91 each, while Capparidaceae achieves an FIV of 0.83, emphasizing its significance as a source of medicinal and food plants. Ultimately, the research suggests sustainable utilization of medicinal plants such as *Ephedra gerardiana*, *Capparis spinosa, Nepeta cataria*, and *Astragalus oihorensis*. *Quercus baloot* serving as a primary food source for Markhor and fuel, demands sustainable utilization. Additionally, it is suggested to increase the cultivation of economically valuable species like *Platanus orientalis* and *Juglans regia*, which are highly utilized in the study area.

## Data Availability

All the data collected during the research work have been made available in the manuscript.

## Abbreviations

TEK: Traditional ethnobotanical knowledge
USSR: Union of Soviet Socialist Republics
UR: Use Report
ICF: Informants Consensus Factor
RFC: Relative Frequency of Citation
UV: Use Value
FL: Fidelity Level
FIV: Family Importance Value
MED: Medicinal
AF: Animal Feed
VU: Veterinary Uses
TECH: Technology
HF: Human Food
TIM: Timber
FW: Fuel Wood
SYM: Symbolic
TOX: Toxic
